# Photonic-chemostat engineering for efficient continuous cultivation of cyanobacteria

**DOI:** 10.1039/d5ra09945e

**Published:** 2026-02-25

**Authors:** Mohammad Redwanur Rahman, Md Tabish Noori, Klaus Hellgardt

**Affiliations:** a Department of Chemical Engineering, Imperial College London London SW7 2AZ UK t.noori@imperial.ac.uk; b Brilliant Dyes Ltd, Scale Space 58 Wood Ln London W12 7RZ UK

## Abstract

Optimising continuous phototrophic cultivation remains a major challenge for scalable, energy-efficient cyanobacterial bioprocesses. Here, we combine controlled photophysiology, long-term continuous experimentation, multi-parameter analysis, and batch-derived Monod kinetic modelling to define a precise operational window for *Synechocystis* sp. PCC 6803 under flat-plate photobioreactor (FP-PBR) illumination. Using a fully calibrated FP-PBR platform, we first quantified intrinsic growth limits (*µ*_max_ = 0.081–0.118 day^−1^) across low, moderate, and high irradiance regimes, establishing the illumination-driven growth ceilings that constrain downstream continuous operation. Guided by these kinetic boundaries, continuous cultivation demonstrated that productive steady-state growth emerges only within a narrow regime governed by light intensity (500–700 µmol photons m^−2^ s^−1^), temperature (32–34 °C), and dilution rate (0.12–0.14 day^−1^). Single-parameter and 3D interaction analyses revealed strong coupling between photonic supply, thermal sensitivity, and hydraulic residence time, while multi-factor modelling captured these nonlinear constraints and accurately predicted washout boundaries. Translating these insights into sustainability metrics, the optimised regime supports 0.07–0.125 g L^−1^ day^−1^ of biomass productivity, equivalent to 8.4–15.0 g biomass day^−1^ and 176–315 kJ day^−1^ of chemical energy in a 120 L mini-pilot system. Stoichiometric analysis indicates this corresponds to 15.6–27.6 g CO_2_ day^−1^ sequestered, demonstrating measurable environmental benefit even at a small scale. Together, these results provide a mechanistically grounded, kinetically constrained framework for designing inherently efficient, low-waste, and model-predictive cyanobacterial photobioprocesses aligned with green chemistry and future carbon-neutral manufacturing.

## Abstract 2

A photonic-chemostat framework delivering maximised photon efficiency and stable continuous cyanobacterial growth for sustainable biomanufacturing.

## Graphical abstract

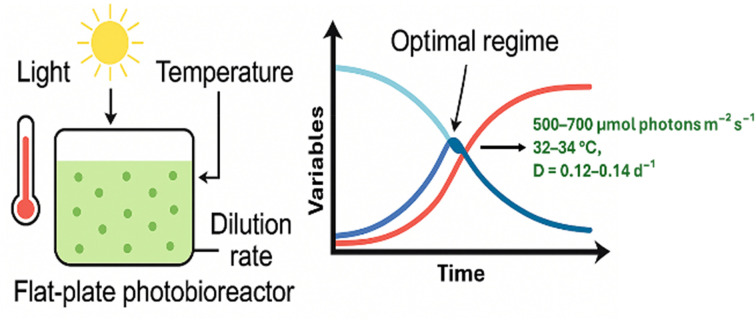

## 1. Introduction

Photosynthetic microbes, such as microalgae and cyanobacteria, have emerged as an efficient biological platform for sustainable biomanufacturing, owing to their ability to convert sunlight, CO_2_, and nutrients into a diverse array of valuable biomolecules.^1–4^ According to the literature, cyanobacteria can capture 1.7–2.0 g CO_2_ per g biomass, with production of 0.45–0.70 g protein per g biomass as a valuable outcome.^4,5^ This intrinsic ability has positioned them at the forefront of global efforts toward carbon-neutral production systems, particularly amid growing concerns over fossil fuel depletion, energy security, and the environmental burden of petrochemical manufacturing.^6^ As global energy demand continues to rise and fossil-derived emissions remain the dominant contributor to climate instability, photosynthetic bioprocesses offer a route to both decarbonisation and circularity by recycling atmospheric carbon into valuable products. Despite these advantages, the commercialisation of photosynthetic biotechnologies has been constrained by system inefficiencies, especially in cultivation and process-intensification strategies within photobioreactors (PBRs), where light delivery, mixing, and nutrient utilisation must be finely coordinated to achieve steady, high-density production.

Among photosynthetic microbes, *Synechocystis* sp. PCC 6803 occupies a unique position as a genetically tractable model cyanobacterium capable of oxygenic photosynthesis, heterotrophic growth, and synthesis of pigments, fatty acids, esters, terpenoids, and other bioactive compounds.^7,8^ This model cyanobacteria is metabolically versatile, using the Calvin–Benson–Bassham (CBB) cycle for CO_2_ fixation and a branched tricarboxylic acid cycle (TCA)/oxidative pentose phosphate network for central carbon flux. This flexibility has positioned *Synechocystis* sp. PCC 6803 as a key platform for renewable biofuels, fine chemicals, nutraceutical precursors, and high-value pigments.^9^ However, while engineered strains have demonstrated proof-of-concept production of esters, free fatty acids, alcohols, and other high-value compounds, their scalability remains hindered by low titres, energy-intensive downstream processing, and instability under continuous operation.^9,10^ Even in wild-type strains, achieving reliable and energy-efficient biomass and pigment production requires an intimate understanding of how physical and biochemical parameters interact within a PBR environment. Light intensity, spectrum, temperature, dilution rate, and internal pH dynamics collectively define the culture's metabolic state, yet these variables often fluctuate unpredictably in laboratory and industrial systems. For instance, a recent study noticed that light–dark regimes and CO_2_ level modulate central metabolism and carbon partitioning in *Synechocystis* sp. PCC 6803, triggering rapid shifts in metabolite pools, growth, and storage-product allocation; true transitions to photomixotrophy and persistent stress-like reprogramming, however, require the presence of exogenous organic carbon and depend strongly on light regime, CO_2_ magnitude, and strain background.

The complex photophysiology of cyanobacteria magnifies this challenge. Light drives ATP and NADPH generation through linear and cyclic electron transfer, but the relationship between photon flux and biomass accumulation is non-linear.^11^ At low irradiance, growth is limited by photon availability, whereas at moderate irradiance, cultures can reach a saturation plateau, and at high irradiance, cells experience photostress, requiring substantial metabolic resources for repair and dissipation. The spectral composition of light further shapes these processes, as cyanobacterial antenna complexes absorb distinct wavelengths with varying quantum efficiencies.^12^ Yet many bench-scale PBRs deliver light non-uniformly, with substantial differences in spectral output and Photosynthetically Active Radiation (PAR) efficiency between units.^6,13^ Such variability introduces significant uncertainties in experimental reproducibility and often leads to misleading assumptions about optimal cultivation conditions.

Moreover, the translation of batch-optimised conditions into continuous culture systems remains poorly understood for cyanobacteria.^14^ Continuous phototrophic cultivation promises stable, cost-effective production by maintaining cells at pseudo-steady state, enabling consistent product quality and reduced downtime.^15^ However, defining operational windows that avoid washout while preventing photoinhibition or nutrient limitation is non-trivial. Most existing literature relies on simplified chemostat models that assume light-limited, single-factor growth kinetics, which do not adequately capture the multidimensional interactions observed in real PBR environments.^16–19^ As a result, continuous cyanobacterial cultivation has often been observed to be unstable over long periods, with productivity losses linked to dilution-rate shocks, light oversupply, temperature fluctuations, and pH oscillations caused by CO_2_ supplementation dynamics.^20,21^ A critical gap, therefore, exists in establishing an integrated, experimentally validated framework that links: (i) empirical spectral characterisation of PBR systems, (ii) multivariate optimisation of light, temperature, and nutrient supply through controlled batch studies, and (iii) long-term continuous cultivation supported by mechanistically grounded growth kinetics. Addressing this gap is essential not only for improving the robustness and energy efficiency of cyanobacterial bioprocesses but also for enabling environmentally sustainable production pathways that are competitive with current petrochemical methods. *Synechocystis* sp. PCC 6803 was selected as a physiologically and genetically well-characterised model cyanobacterium, widely used in studies of photosynthesis, metabolism, and photobioreactor operation, enabling mechanistic interpretation of operating-window boundaries under controlled conditions.^22,23^ The flat-panel photobioreactor was used as a highly controlled scale-down platform to generate transferable stability and control rules, rather than to represent a specific industrial configuration. The 120 L analysis is therefore presented as a mini-pilot translation of productivity metrics, intended to demonstrate the scalability of the operating-window concept, rather than as a claim of full industrial deployment.

In this study, we develop a calibration-integrated framework for defining the coupled operating window of continuous cyanobacterial cultivation. We combine spectral characterisation of flat-panel photobioreactors with data-driven batch photophysiology and a 90 day continuous cultivation of *Synechocystis* sp. PCC 6803 (hereafter PCC 6803). Using calibrated reactors, we quantify how intensity-dependent spectral shifts and photon delivery influence biomass accumulation and stability. A multivariate experimental design is then used to examine the coupled effects of light intensity, temperature, and dilution rate, enabling identification of the stability boundaries governing continuous operation. Finally, we implement a simplified kinetic framework linking batch-derived growth behaviour to steady-state continuous performance, allowing prediction of washout risk and operating limits. Together, these results establish a mechanistically grounded approach for defining kinetically constrained operating windows in phototrophic chemostats, providing transferable design principles for stable and energy-efficient photobioprocess operation.

## 2. Materials and methods

### 2.1. Strains and media preparation

Wild-type PCC 6803 was obtained from the Pasteur Culture Collection of Cyanobacteria (PCC, Institut Pasteur, France) and maintained on BG11 agar plates under continuous LED illumination within a CO_2_ incubator. Liquid cultures were initiated by transferring single colonies into a 6-well plate, then into 50 mL and 250 mL Erlenmeyer flasks containing sterile BG11 medium, and grown photoautotrophically at 30 °C. BG11 was prepared according to the standard composition, containing NaNO_3_ as the nitrogen source, K_2_HPO_4_ as the phosphorus source, a trace metal solution, and bicarbonate-free base salts (SI, S1). All media were sterilised by autoclaving at 121 °C for 20 min, while ferric ammonium citrate and Na_2_CO_3_ were filter sterilised separately and added aseptically after cooling. Pre-cultures were grown to mid-exponential phase (OD_680_ 0.6–0.8) before inoculation into batch or continuous PBR.

For continuous experiments, the feed medium was prepared identically to BG11, supplemented with 2% (v/v) phosphate buffer, and aerated with 3% (v/v) CO_2_-enriched air to stabilise pH fluctuations observed during long-term cultivation. Media reservoirs, overflow bottles, tubing, and connectors were sterilised by autoclaving immediately before each experimental run.

### 2.2. FP-PBR setup and spectral calibration methods

**Fig. 1:**
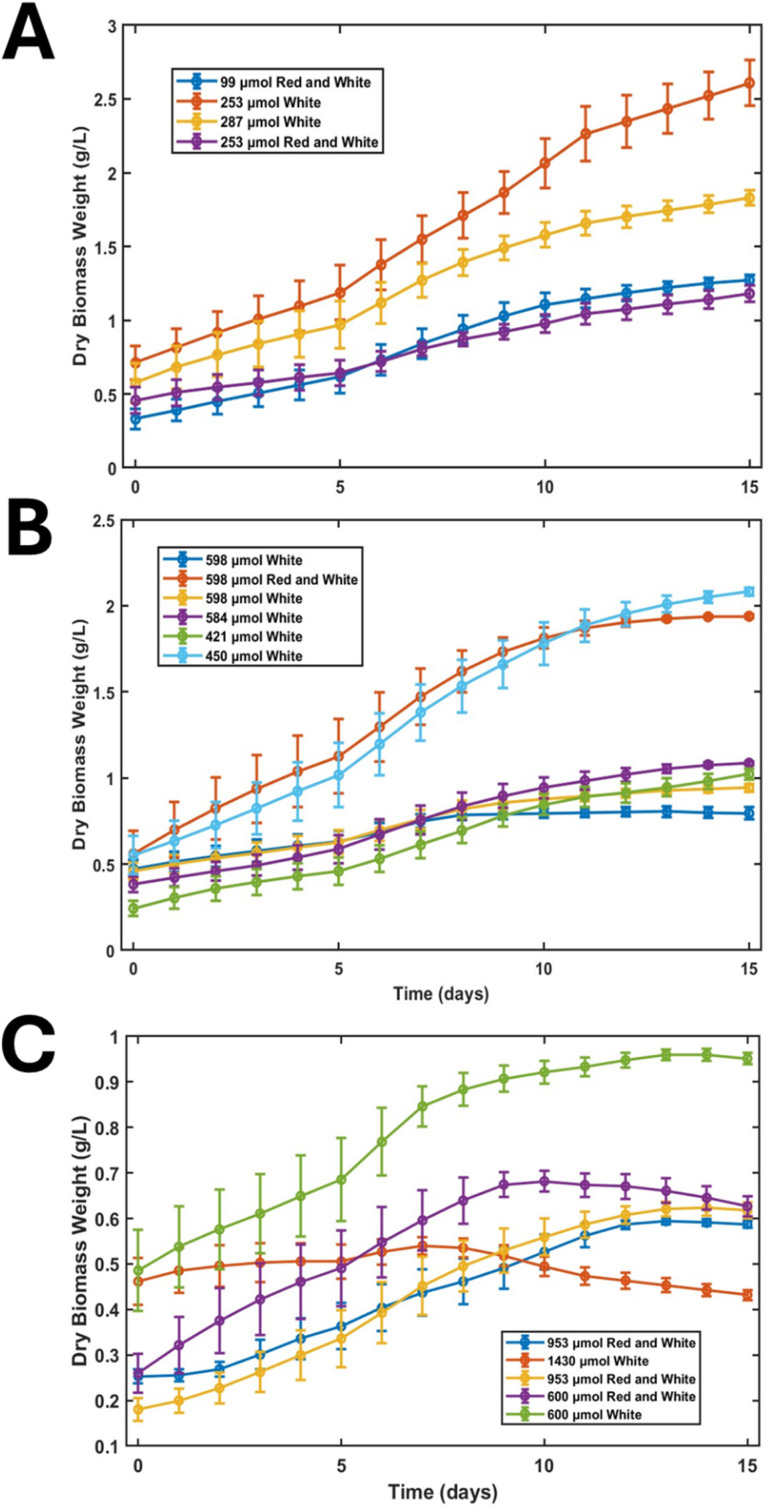
Batch growth studies at different light conditions: (A) low (99–300 µmol photons m^−2^ s^−1^), (B) moderate (300–599 µmol photons m^−2^ s^−1^), and (C) high (600–1430 µmol photons m^−2^ s^−1^).

Batch and continuous experiments were conducted using the PSI FMT-150 flat-panel photobioreactor (Photon Systems Instruments, Czech Republic) equipped with integrated LED illumination panels, temperature regulation, gas mixing, and online optical density monitoring (SI, Fig. 1S). Each FP-PBR consisted of a 400 mL working volume vessel with internal baffles to enhance light scattering and mixing. Temperature was regulated using a built-in thermoregulator connected to a circulating water bath, allowing control of a wide temperature range between 5–75 °C; however, in this study, a temperature between 28–36 °C was studied. Gas flow was maintained at 0.2–0.3 vvm using a mass-flow controller, delivering humidified air enriched with 2–3% CO_2_ (v/vol in air), depending on the experimental phase. In continuous cultures, pH fluctuations between 6.0 and 10.5 were automatically monitored and recorded in the data logger connected to the personal computer.

LED illumination intensities were calibrated externally using a high-precision spectroradiometer (Thorlabs CCS200/M/M) before all experiments. Each PBR was characterised individually to account for observed inter-reactor variability in spectral distribution and output intensity. Measurements included full emission spectra (350–800 nm), incident photon flux density (PPFD, µmol m^−2^ s^−1^), wavelength-integrated PAR efficiency, and spatial uniformity across the cultivation window. Calibration confirmed substantial spectral differences between reactors, especially under white and combined red + white illumination regimes. This ensured that all reported PPFD values reflect actual incident light levels rather than manufacturer-set intensities, and that their performance or efficacy does not change over time. Because spatial non-uniformity is intrinsic to illuminated photobioreactors, calibration is not intended to eliminate photon gradients but to quantify the true incident PPFD and spectral composition delivered to the culture. This step prevents optical offsets between reactor modules from being misinterpreted as biological effects. All reported PPFD values therefore correspond to externally measured photon flux at the cultivation window, and the intensity-dependent spectral shift of each LED module is treated as an experimentally defined property of the illumination system when interpreting growth responses.

### 2.3. Batch and continuous experimental design

A total of 15 custom-designed batch photophysiology trials at low (<300 µmol m^−2^ s^−1^), moderate (300–599 µmol m^−2^ s^−1^), and high (>600 µmol m^−2^ s^−1^) LED intensities were performed to determine the independent effects of PPFD and light spectrum on biomass accumulation. Triplicate 400 mL FP-PBRs were inoculated to OD_680_ = 0.6–0.8, and each was grown for 15 days under controlled temperature (30 °C), gas supply (3% CO_2_), and defined white or red + white illumination. Light intensity was maintained at a constant PPFD (µmol m^−2^ s^−1^) throughout each 15 day batch trial, and data were monitored at 10 second intervals using the online sensors in the FMT150 PBR. Real-time data, including OD_680_, OD_720_, pH, and temperature, has been recorded using the FMT150 software. Key growth phases were used to determine saturation points and the early onset of photostress, enabling parameter selection for continuous trials. Batch experiments were conducted in technical triplicate, and results are reported as mean ± standard deviation. Statistical significance between batch conditions was assessed using appropriate tests after verification of distributional assumptions.

Continuous-mode experiments were initiated on day 1 following batch inoculation. Dilution rates (*D*) were modulated from 0.0375 to 0.20 day^−1^ in a stepwise manner to map washout thresholds and steady-state regions. Light intensity was adjusted step-by-step and studied within 250–800 µmol m^−2^ s^−1^ in response to biomass changes, while temperature was progressively increased from 30 to 36 °C over the 90 day operation to assess thermal tolerance in a long-term chemostat regime. Biomass was harvested continuously *via* a sterile overflow system. Fresh medium was supplied using a calibrated peristaltic pump (±0.2% accuracy). System integrity and sterility were verified daily and adjusted as needed to conduct the prolonged, continuous experiment. The 90 day continuous cultivation was designed as an operating-window mapping experiment rather than a single-point optimisation. Stepwise adjustments of dilution rate, light intensity, and temperature were implemented to avoid shock-induced collapse while progressively approaching stability boundaries and to emulate incremental tuning practices commonly used in pilot-scale operations.

Selective single-factor and multifactor experiments were extracted from the continuous dataset. Conditions were screened for intervals in which only one parameter (light intensity, temperature, or dilution rate) was varied while the others remained constant. These data subsets were used to evaluate individual parameter effects and to construct 3D response surfaces.

### 2.4. Analytical methods and calculations

**Fig. 2:**
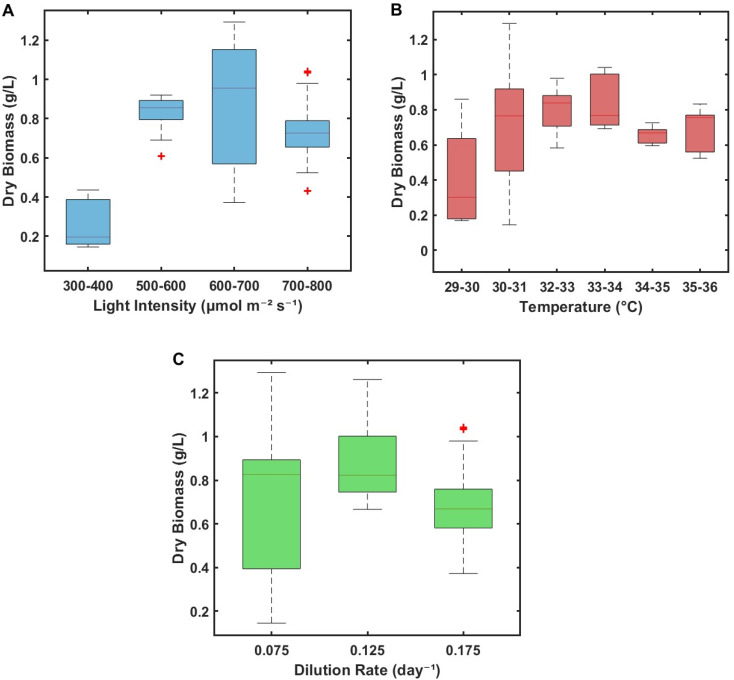
Investigation of the individual effects of changing light intensity, temperature and dilution rate on the relative dry biomass concentration (A) effects of changing light intensity on dry biomass concentrations when the temperature and dilution rate are maintained at 30 ± 0.3 °C and 0.075 ± 0.006, respectively, (B) effects of changing temperature between 29–36 °C when the light intensity and dilution rate were maintained between 600–700 µmol photons m^−2^ s^−1^ actinic white and 0.075 ± 0.006, respectively. (C) Effects of changing media dilution rates of 0.075 ± 0.006, 0.125 ± 0.008 and 0.175 ± 0.009 on the biomass production when the light intensity and temperature are maintained between 600–700 µmol photons m^−2^ s^−1^ actinic white and 30 ± 0.3 °C, respectively.

Optical density at 680 nm was measured using both the FP-PBR online detector and a calibrated UV-vis spectrophotometer (Shimadzu UV-2600). Dry cell weight (DCW) was calculated using a standard OD–DCW calibration curve established before experiments (SI, Fig. 2S). Biomass samples (5–10 mL) were centrifuged, washed twice with deionised water, and dried at 50 °C for 12 h. Temperature, pH, and dissolved oxygen were logged every 10 minutes using the PBR's integrated sensors. A mass flow controller monitored the CO_2_ flow rate. Light intensity during operation was periodically verified using an external quantum sensor (Apogee MQ-500). For continuous cultures, steady-state was assessed when biomass concentration and OD_680_ varied by <5% over three residence times. All measurements were performed in technical triplicate unless stated otherwise.

For energetic and photosynthetic efficiency analyses, volumetric biomass productivity (g L^−1^ day^−1^), incident photon flux (µmol m^−2^ s^−1^), and biomass higher heating value were used to estimate chemical energy storage, photon-to-biomass conversion efficiency, and CO_2_ sequestration. Stoichiometric assumptions, reactor-geometry corrections, and all equations used to calculate photosynthetic efficiency, photon energy flux, and energy recovery ratios are detailed in SI (S2). These calculations were applied only to steady-state continuous cultures.

### 2.5. Kinetic model fitting workflow

A 15 day batch cultivation dataset was used to parameterise a Monod-type growth kinetic model under low, moderate, and high illumination regimes. Biomass concentration (g L^−1^) was recorded daily from day 0 to day 15. For kinetic analysis, only the exponential growth region (day 3–10) was used to ensure that modelling reflected intrinsic growth rather than early adaptation or late nutrient limitation.

Growth kinetics were represented using the following Monod eqn (1). The dilution rate, *D* (day^−1^), is defined as the inverse of the hydraulic residence time imposed during continuous operation, whereas *µ* (day^−1^) represents the biological specific growth rate estimated from batch exponential-phase fits or inferred relative to *D* under steady-state continuous conditions.1
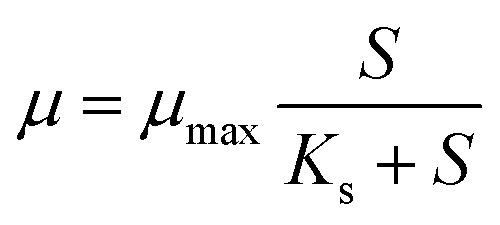


Because phototrophic cultures in BG-11 are not substrate-limited during early batch growth, the substrate concentration *S* was assumed to remain non-limiting, and *K*_s_ was fixed at 0.10, consistent with literature values for *Synechocystis* kinetic fitting. Under these conditions, biomass follows (eqn (2)).

2 *X*(*t*) = *X*_0_ exp(*µ*_eff_*t*)

where *µ*_eff_ is the effective observed specific growth rate derived from Monod behaviour.

The Monod-type framework used in this study is intentionally simplified and applied primarily to the exponential batch region to estimate an effective kinetic ceiling under defined illumination regimes. Nonlinear stress phenomena such as photoinhibition, reactive oxygen species formation, and pH-dependent physiological limitations are not explicitly incorporated as inhibition terms. Instead, their effects are implicitly reflected in the reduced effective growth behaviour observed under high-light or high-pH conditions. As a result, the model is most applicable within the non-photoinhibitory operating window and for predicting washout boundaries under comparable physiological states, while extrapolation to extreme stress regimes would require an extended model formulation including explicit inhibition terms.

For each illumination condition, the model was fitted to the experimental biomass trajectory using nonlinear least-squares regression implemented in MATLAB (SciPy optimize.minimize). Three parameters were estimated: (i) *µ*_max_ (maximum specific growth rate), (ii) *K*_s_ = 0.10 (held constant across all fits), and (iii) *X*_max_ (model-predicted final biomass at day 10). The objective function minimised the sum of squared residuals between observed biomass *X*_exp_(*t*) and model predictions *X*_mod_(*t*). Confidence intervals for fitted parameters were obtained from the Jacobian of the residual space. Model performance was assessed using RMSE, Adjusted *R*^2^, and Residual distribution plots.

## 3. Results and discussion

### 3.1. LED spectrum variability analysis and calibration

Accurate quantification of light input is fundamental to the optimisation of phototrophic bioprocesses, yet LED-driven flat-panel photobioreactors (FP-PBRs) often exhibit substantial variability in photon delivery depending on the specific LED configuration, driving electronics, and spectral output stability.^24,25^ In this study, the FP-PBR platform was operated with multiple interchangeable LED panels—actinic red, actinic white, and combined red–white arrays—to perform a series of parallel customised batch and continuous cultivation experiments. Calibration of the illumination modules using different LED panels (referred to here as PBR1-4) revealed pronounced differences in both absolute spectral intensity and wavelength distribution, despite being controlled at the designed nominal PPFD setpoints.

**Fig. 3:**
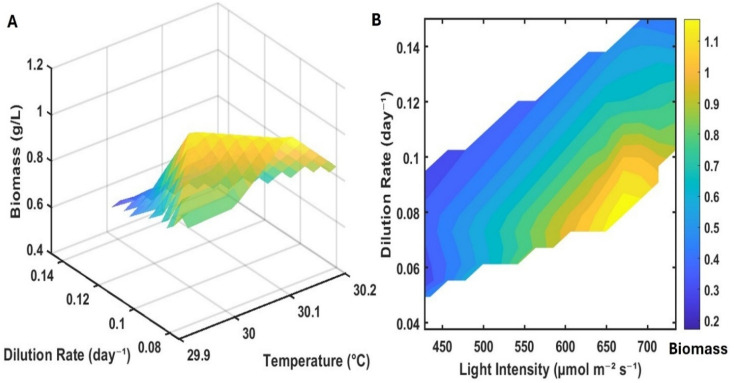
3D contour plot and heatmap showing the cumulative effects of multiple parameters on the relative biomass concentration in a continuous growth trial in the small lab-scale PBR (A) effect of changing dilution rate (0.08–0.14 day^−1^) and temperature (29.9–30.2 °C) on the dry biomass concentrations and (B) Heatmap showing the combined effect of increasing light intensity between 450 to 750 µmol photons m^−2^ s^−1^ actinic white and a dilution rate between 0.05 and 0.15 day^−1^.

Spectral scans (400–750 nm) showed that the actinic red LED configuration produced a strong monochromatic peak centred at 630–640 nm, reaching relative intensities of ∼5600 at high irradiance (900 µmol photons m^−2^ s^−1^) (S1, Fig. 3S). In contrast, actinic white and red–white mixed LED arrays generated broad dichromatic spectra with peaks in the blue (420–460 nm) and green–red (510–600 nm) regions. However, the three LED types differed markedly in total output: at comparable PPFD setpoints, the white LEDs produced ∼3300 in relative intensity, while the red–white combination produced ∼1000–1050. These differences in photon delivery across LED modules demonstrate that light quality and quantity cannot be assumed to be equivalent even when set to the same PPFD, underscoring the need for direct calibration.

Importantly, spectral composition within each LED configuration changed as irradiance increased. For example, using the red–white LED module, the red:blue ratio fell from 0.96 at low irradiance (10 µmol photons m^−2^ s^−1^) to 0.56 at high irradiance (1200 µmol photons m^−2^ s^−1^), indicating a shift toward green-dominant spectra with increasing current. Conversely, the actinic white LED showed far greater spectral stability with only minor shifts in Red–Green–Blue (RGB) proportions, while the actinic red LED exhibited increasing red dominance at higher PPFD (up to ∼90% of total spectral composition at 900 µmol photons m^−2^ s^−1^). These observations confirm that spectral drift is intensity-dependent and that the wavelength composition changes with LED drive level, which can significantly affect cellular excitation balance and photophysiological responses.^26^

**Fig. 4:**
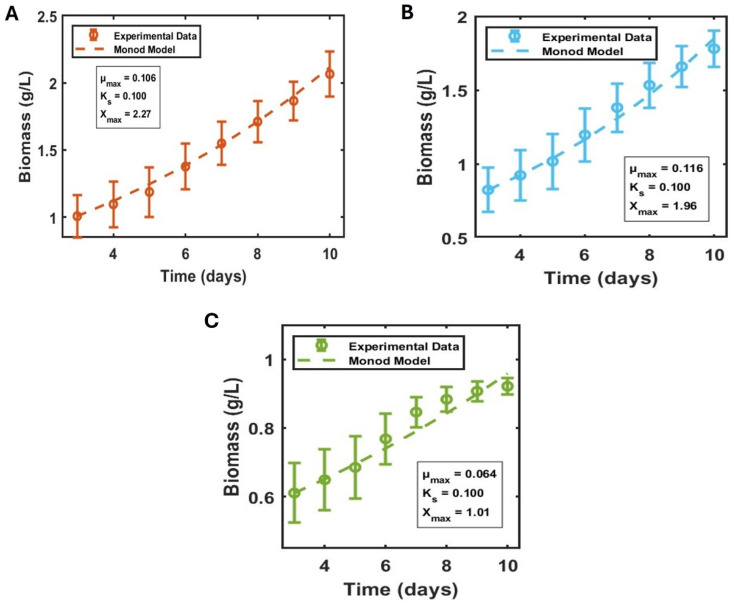
Representative best growth experiments from (A) low, (B) moderate, and (C) high light intensity experiments' kinetic model fitted and compared with the theoretical Monod model.

Calibration curves comparing PPFD setpoints to measured photon flux showed that the set values did not match the actual PPFD delivered at the culture surface (SI, Fig. 4S). The deviations were large enough that each LED configuration required an independent calibration curve. Notably, the slope and linearity of the PPFD response differed markedly between the actinic red, actinic white, and red–white combined LEDs. Without applying these correction curves, subsequent growth responses, particularly comparisons across low, moderate, and high light regimes, would risk being misinterpreted as biological variability rather than illumination artefacts.^24^ The results, therefore, emphasise that accurate optical calibration is essential when using LED-based PBR systems with multiple illumination modes. The substantial variation in photon flux and spectral composition across LED types, combined with intensity-dependent spectral drift, highlights the importance of quantifying the true light environment before biological experimentation. The calibrated PPFD and spectral profiles obtained here formed the basis for all subsequent batch and continuous cultivation analyses.

### 3.2. Batch growth optimisation at different spectral intensities

Batch cultivation experiments under controlled LED illumination were first performed to establish the practical photophysiological limits of the wild-type *Synechocystis* sp. PCC 6803 prior to designing and operating continuous photobioreactor systems. The wild-type strain was initially maintained on BG-11 agar plates to ensure purity and stable colony morphology (SI, Fig. 5S). Cells recovered from agar plates and transitioned into suspension cultures exhibited rapid acclimation, reaching stable exponential growth within 24–36 hours. UV-vis spectral analysis of the suspended cultures showed distinct absorption peaks characteristic of healthy cyanobacterial photopigments, with chlorophyll a displaying a strong Soret band at ∼430 nm and a red absorption band of chlorophyll molecules (*Q*_y_) peak at ∼680 nm, while phycocyanin exhibited its typical absorption maximum around ∼620 nm (SI, Fig. 5S). The stable pigment profile confirmed active photosynthetic machinery and the absence of bleaching or stress. These well-acclimated cultures were subsequently used for all further tests and continuous photobioreactor trials.

Across a series of customised trials spanning low (99–299 µmol photons m^−2^ s^−1^), moderate (300–599 µmol photons m^−2^ s^−1^), and high (>600 µmol photons m^−2^ s^−1^) light intensities, growth behaviour was strongly governed by both photon flux and spectral composition, with white-light regimes consistently outperforming red–white combinations.

Under low-light conditions (99–287 µmol photons m^−2^ s^−1^), cultures displayed a pronounced 2–3 day lag phase with slowly increasing OD_680_, reflecting acclimation to limited photon availability. Exponential growth commenced around day 3, but with relatively shallow slopes compared with higher light regimes. Final dry biomass ranged from 1.1 to 3.0 g L^−1^, with the highest value obtained at 253 µmol photons m^−2^ s^−1^ actinic white light, whereas red–white combinations at similar intensities produced notably lower biomass (Fig. 1A). Specific growth rates remained modest (0.15–0.45 day^−1^), consistent with literature reports for PCC 6803 in batch culture.^14,27^ Throughout these trials, temperature remained stable at 30 ± 0.7 °C and pH increased gradually from 7.0 to around 9.2, indicating active carbon fixation without signs of photoinhibition or severe stress. These observations suggest that at low light, cells prioritise acclimation and optimisation of light-harvesting capacity,^28–30^ with white spectra at intensities above ∼250 µmol photons m^−2^ s^−1^ enabling robust yet non-stressed growth. This is consistent with previous findings that balanced white-spectrum illumination promotes more uniform excitation of PSI and PSII, improving early-stage carbon fixation relative to red-dominant spectra.^26^ In our experiments, biomass accumulation in the low-light regime remained modest yet highly reproducible, indicating that cultures were predominantly photon-limited.

At moderate light intensities (300–599 µmol photons m^−2^ s^−1^), growth became more heterogeneous and less reproducible, as was also reported earlier.^18^ Across six parallel trials, cultures commonly exhibited a 1–2 day lag, followed by a quasi-linear increase in OD_680_ rather than a smooth exponential phase (Fig. 1B). In several experiments (421–598 µmol photons m^−2^ s^−1^, mainly white light), biomass plateaued around 0.7–1.1 g L^−1^ by day 10 and subsequently declined (Fig. 1B), indicating the onset of light stress rather than sustained productive growth. Two conditions stood out (i) 450 µmol photons m^−2^ s^−1^ actinic white and (ii) 598 µmol photons m^−2^ s^−1^ actinic red and white with a dominant white component. These trials reached ∼2.0 g L^−1^ by day 10, with the 450 µmol photons m^−2^ s^−1^ white experiment continuing to increase to ∼2.2 g L^−1^ by day 15, representing the best overall performance in the moderate-intensity range. In contrast, 421 µmol photons m^−2^ s^−1^ white achieved only ∼0.85 g L^−1^ by day 10, indicating that this intensity remained effectively light-limiting. Environmental conditions remained controlled (30.2 ± 0.3 °C), while pH fluctuated more widely between 7.5 and 10.8, reflecting more vigorous metabolic activity and more complex carbon assimilation dynamics under moderate light.

In the high-light regime (>600 µmol photons m^−2^ s^−1^), five customised trials revealed clear thresholds for photoinhibition. Increasing irradiance beyond approximately 600 µmol photons m^−2^ s^−1^ did not improve biomass formation; instead, cultures experienced extended lag phases, low and unstable exponential growth, and, in many cases, collapse after 8–10 days. The best-performing high-light condition, 600 µmol photons m^−2^ s^−1^ actinic white, reached only ∼0.95–1.0 g L^−1^ by day 10, substantially lower than the yields obtained at 253 or 450 µmol photons m^−2^ s^−1^. Other high-light trials (600 µmol photons m^−2^ s^−1^ red–white; 953 and 1430 µmol photons m^−2^ s^−1^) failed to exceed ∼0.4–0.65 g L^−1^ and often showed visible bleaching and chlorosis within 5–7 days, indicative of photodamage. Specific growth rates declined markedly compared with the low- and moderate-trial conditions, and pH frequently rose above 9.0, reaching>10.0 in some experiments (Fig. 1C). The pH excursions into the range of 9–10 observed under high irradiance are consistent with rapid inorganic carbon uptake by the culture. Under elevated photon flux, the rate of photosynthetic CO_2_ assimilation can temporarily exceed the CO_2_ mass-transfer rate from the gas phase, even when a 3% CO_2_-enriched stream is supplied. This imbalance shifts the carbonate equilibrium toward alkalisation, resulting in elevated pH values. Such conditions can impose physiological stress through reduced CO_2_ availability, altered membrane energetics, and changes in metal speciation, which together contribute to the decline in stability and productivity observed at high irradiance. The pH is therefore interpreted as a coupled state variable within the operating window rather than a passive measurement. High irradiance leads to PSII overexcitation, impaired linear electron flow, and accumulation of reactive oxygen species.^31,32^ Additionally, carotenoid-to-chlorophyll ratios increased under high-light conditions, with cultures often displaying visible chlorosis by day 5–7, a hallmark of photo-oxidative stress.^33^ In parallel, the pH frequently rose beyond the desired window, exceeding pH 10 in several cases despite CO_2_ supplementation, reflecting rapid alkalisation driven by intense photosynthetic carbon uptake – another strong indicator of metabolic stress.^34,35^ Moreover, in the 600 µmol photons m^−2^ s^−1^ white-light trial, pH climbed near 12 by day 5 before declining later, consistent with strong CO_2_ uptake under stress and impaired downstream utilisation. Temperature remained largely stable (30.3 ± 1.5 °C) except under the extreme 1430 µmol photons m^−2^ s^−1^ treatment, where additional heating from the LEDs required active correction, confirming that the inhibition observed was primarily photophysiological rather than thermal. The reduced stability observed at high irradiance can be explained by known photophysiological mechanisms in cyanobacteria. Under excessive photon flux, excitation pressure on photosystem ii can exceed the capacity of downstream electron transport and carbon fixation pathways, leading to photoinhibition and increased formation of reactive oxygen species.^36^ At the same time, rapid CO_2_ assimilation shifts the carbonate equilibrium, driving alkalisation of the medium and reducing the availability of dissolved inorganic carbon.^37^ The combined effects of photoinhibition, oxidative stress, and elevated pH therefore provide a mechanistic explanation for the observed decline in biomass stability and productivity at high irradiance.

Batch experiments show that photon flux and spectral quality jointly define a relatively narrow operational window for productive, non-stressed growth of *Synechocystis* sp. PCC 6803. White-light illumination at ∼250–450 µmol photons m^−2^ s^−1^ consistently supported the highest and most stable biomass accumulation, while lower intensities produced slower but healthy growth, and intensities above ∼500–600 µmol photons m^−2^ s^−1^ induced significant adaptation burdens, reduced final biomass, and, at very high levels, culture collapse. Those results are consistent with previous studies on *Synechocystis* sp. PCC 6803 shows that both light intensity and spectral composition critically shape photophysiological performance, with moderate irradiance supporting efficient energy transduction while excessive photon flux leads to redox imbalance and stress.^38^ The observed decline in biomass above ∼500–600 µmol photons m^−2^ s^−1^ reinforces the concept that phototrophic growth operates within a narrow window defined by the balance between photon supply and the metabolic capacity to utilise photosynthetically generated ATP and NADPH.^39^ It is important, however, to distinguish between endpoint batch biomass and steady-state continuous operability. The higher final biomass observed at low irradiance reflects sustained low-stress accumulation over a prolonged cultivation period. In continuous cultivation, however, stability requires that the effective growth rate remains higher than the dilution rate while avoiding stress-induced productivity loss. Consequently, batch conditions that yield high endpoint biomass do not necessarily correspond to the most stable or productive continuous operating points. These findings are in agreement with recent semi-continuous cultivation studies on *Chlorella sorokiniana*, demonstrating that intermediate dilution fractions maximise long-term productivity and compositional stability.^40^ Our results similarly show that sustained performance depends on maintaining a balance between growth kinetics and hydraulic removal rather than maximising endpoint biomass. Together, these findings reinforce that stable continuous or semi-continuous operation is governed by a constrained operational window in which dilution rate must remain tightly coupled to physiological growth capacity to avoid productivity loss or washout.

### 3.3. Transition to continuous operation

To translate the batch-optimised conditions into a robust continuous regime, the 90 day experiment was dissected into selective data segments in which only one key parameter—light intensity, temperature, or dilution rate—was varied while the others were held within a narrow range. This approach allowed the intrinsic operating window of *Synechocystis* sp. PCC 6803 in continuous mode to be quantified without the confounding effects of simultaneous multi-parameter changes. Full-time course profiles of biomass, dilution rate, light intensity, temperature, and pH are provided in the SI (Fig. 6S), while here we focus on the steady-state trends extracted from that dataset.

The effect of light intensity was evaluated over a broad range from 300 to 800 µmol photons m^−2^ s^−1^ actinic white, at a fixed temperature of 30 °C and dilution rate of 0.075 d^−1^ (Fig. 2A). At 300–400 µmol photons m^−2^ s^−1^, biomass concentrations remained low (0.2–0.4 g L^−1^), reflecting limited photon availability and relatively early-stage culture density. Increasing PPFD to 500–600 µmol photons m^−2^ s^−1^ substantially enhanced performance, with biomass rising to 0.8–0.9 g L^−1^. A further increase to 600–700 µmol photons m^−2^ s^−1^ yielded a wider spread of 0.6–1.1 g L^−1^, highlighting the biological variability inherent in continuous phototrophic systems even when nominal conditions are fixed. Beyond this range, at 700–800 µmol photons m^−2^ s^−1^, biomass declined to ∼0.65–0.75 g L^−1^. Taken together, these results identify 500–700 µmol photons m^−2^ s^−1^ actinic white as the most suitable light-intensity window for continuous PCC 6803 cultivation: low enough to avoid severe photostress, yet high enough to support elevated volumetric productivity. The observations of this study closely align with previous turbidostat studies by Cordara *et al.*, showing that *Synechocystis* growth increases with irradiance up to ∼500 µmol photons m^−2^ s^−1^, with photoinhibitory effects emerging near 800 µmol photons m^−2^ s^−1^.^41^ Similarly, the optimal 500–700 µmol photons m^−2^ s^−1^ window identified here reflects a balance between enhanced photosynthetic activity and the onset of light-induced stress, further supporting the importance of finely regulating incident irradiance to stabilise continuous photobioreactor performance. The data points shown represent steady-state windows extracted from segmented intervals of a single long-term continuous culture rather than independent replicate runs; variation therefore reflects physiological state transitions, optical drift, and pH coupling effects across the operating window.

The temperature dependence of continuous growth was probed by varying temperature between 29 and 36 °C while maintaining light intensity and dilution rate between 600–700 µmol photons m^−2^ s^−1^ and 0.075 d^−1^, respectively (Fig. 2B). Within 29–31 °C, biomass concentrations ranged from 0.2 to 0.91 g L^−1^. Raising the temperature to 32–34 °C improved performance, with biomass stabilising at 0.7–1.0 g L^−1^. Further increasing the temperature to 34–36 °C reduced biomass to 0.58–0.71 g L^−1^, despite the same light and dilution conditions. Thus, under continuous operation, PCC 6803 exhibits an apparent optimum around 32–34 °C: slightly above the standard 30 °C laboratory setting but below the higher temperatures at which productivity begins to decline. These findings are consistent with the reported thermal tolerance of PCC 6803, which can survive at higher temperatures but does not necessarily achieve higher biomass yields under such conditions.

The influence of dilution rate was then examined at fixed light and temperature (600–700 µmol photons m^−2^ s^−1^, 30 °C; Fig. 2c). Within the tested window of 0.075–0.175 d^−1^, a distinct optimum emerged. At *D* = 0.075 d^−1^, biomass remained modest, reflecting conservative harvesting and a relatively low throughput. Increasing *D* to 0.125 d^−1^ produced the highest and most stable biomass, consistently in the range 0.75–1.0 g L^−1^. Pushing the dilution rate to 0.175 d^−1^ caused biomass to drop sharply, approaching washout. Dilution rates below 0.075 d^−1^ did not offer practical productivity benefits, whereas rates above 0.175 d^−1^ compromised culture stability. Thus, a narrow operational window of 0.12–0.14 d^−1^ emerges as the practical compromise between maintaining high biomass concentration and preventing washout, in line with continuous-culture theory and previous cyanobacterial chemostat studies.

To capture how these parameters interact, the single-factor analyses were complemented by 3D response surfaces constructed from the same 90 day dataset (Fig. 3). The dilution–temperature surface showed that the highest biomass concentrations were obtained at dilution rates between 0.09 and 0.14 d^−1^ and a temperature slightly above 30 °C, whereas minor deviations in either variable led to pronounced declines (Fig. 3A). In parallel, the light–dilution heatmap revealed that biomass above ∼1.1 g L^−1^ was observed only at light intensities of 650–700 µmol photons m^−2^ s^−1^ and dilution rates between 0.07 and 0.09 d^−1^ (Fig. 3B). At higher dilution rates, even under the same high-light conditions, biomass dropped substantially.

The results show that continuous PCC 6803 cultivation does not have independent “best” values for light, temperature, or dilution rate. Instead, productive operation is confined to a small, intersecting region of parameter space: 500–700 µmol photons m^−2^ s^−1^, 32–34 °C, and *D* ∼0.12–0.14 d^−1^. Operating outside this joint window—by increasing light, temperature, or dilution rate in isolation—does not enhance productivity but instead increases the risk of washout or physiological stress. The numerical boundaries of the operating window identified here are specific to the physiology of *Synechocystis* sp. PCC 6803, the flat-panel geometry, and the spectral characteristics of the LED system employed. Different cyanobacterial strains or reactor configurations may exhibit shifts in these boundaries due to variations in light tolerance, temperature sensitivity, or carbon uptake capacity. However, the underlying principle that continuous phototrophic systems operate within a narrow, kinetically constrained window defined by the interaction of photon supply, temperature, dilution rate, and pH dynamics is expected to be broadly applicable.

The calibrated operating-window analysis presented here is intended to improve interpretability and reproducibility rather than to claim a direct increase in absolute biomass yield. The steady-state biomass concentrations and productivity ranges observed in this study are comparable to values reported for continuous cultivation of *Synechocystis* sp. PCC 6803, where biomass productivities of approximately 0.15–0.30 g L^−1^ day^−1^ have been reported under controlled illumination conditions.^42,43^ The primary advantage of the present approach, therefore, lies in defining a reproducible, kinetically constrained operating window and identifying stability and washout boundaries under calibrated illumination, rather than in achieving a single maximum yield value.

### 3.4. Integrated multi-parameter analysis and kinetic modelling of continuous growth

Batch cultivation under fifteen illumination regimes enabled a systematic evaluation of the intrinsic growth kinetics of *Synechocystis* sp. PCC 6803 and the derivation of Monod-based parameters describing photon-driven biomass accumulation. The kinetic responses were grouped into three illumination categories—low (99–287 µmol photons m^−2^ s^−1^), moderate (421–598 µmol photons m^−2^ s^−1^), and high (600–1430 µmol photons m^−2^ s^−1^). Across all conditions, PCC 6803 entered exponential growth between days 3 and 10, allowing robust fitting of the Monod model. This approach has been widely used for cyanobacterial kinetic characterisation under light-variable conditions.^44^Fig. 4 shows the representative biomass production kinetic model plot for each illumination condition at which the biomass growth was noted highest, such as 253 µmol m^−2^ s^−1^ actinic white (Fig. 4A), 450 µmol m^−2^ s^−1^ actinic white (Fig. 4B), and 600 µmol m^−2^ s^−1^ actinic white (Fig. 4C).

Under low illumination, biomass production increased modestly from ∼0.4–0.7 g L^−1^ on day 3 to ∼1.0–2.1 g L^−1^ by day 10. The fitted *µ*_max_ for the representative curve (Fig. 4A) was 0.106 day^−1^, with an *X*_max_ of 2.27, consistent with photon-limited growth. All other low-light replicates (SI, Fig. 7S) displayed similarly shallow slopes, with *µ*_max_ remaining below 0.1 day^−1^ across the 99–287 µmol m^−2^ s^−1^ range, with *X*_max_ value ranging 1.2–1.7. These values align closely with literature reporting *µ*_max_ = 0.06–0.09 day^−1^ for PCC 6803 under sub-saturating light.^45^ The consistency across replicates indicates that in this range, photon supply is insufficient to drive maximal carbon assimilation, even when nutrient and temperature conditions remain favourable.

A clear transition into light-saturated behaviour emerged under moderate light intensity illumination, and under this condition, biomass production increased modestly from ∼0.3 g L^−1^ to ∼0.8 g L^−1^ on day 3 to 1.85 g L^−1^ by day 10. The representative curve (Fig. 4B) showed the highest biomass growth to ∼1.85 g L^−1^, with a fitted *µ*_max_ of 0.116 day^−1^ and *X*_max_ of 1.96, the highest observed across all categories. Other conditions within the 421–598 µmol m^−2^ s^−1^ range (SI, Fig. 8S) exhibited *µ*_max_ between 0.05–0.116 day^−1^ and *X*_max_ values spanning 0.87–1.96 g L^−1^, demonstrating a consistent kinetic enhancement relative to low light. This *µ*_max_ range closely matches reported optimal values for PCC 6803 under non-photoinhibitory white light (0.10–0.13 day^−1^),^46^ confirming that this illumination window provided near-optimal excitation of the photosynthetic apparatus. Moderate-light conditions produced the steepest exponential growth trajectories and the lowest model residuals, further indicating that this regime is physiologically favourable for wild-type PCC 6803.

The highlight condition demonstrated complex growth behaviour; however, the biomass production on day 3 ranged from 0.25 to 0.06 g L^−1^, also showing growth in the lag phase, the least among the tested conditions. The maximum biomass growth, however, reached from 0.42 to 0.96 g L^−1^. The representative curve (Fig. 4C) showed an overall biomass yield of ∼0.96 g L^−1^, but with slightly lower *µ*_max_ (0.064 day^−1^) and *X*_max_ (1.01) than in low or moderate light. Across the full highlight intensity conditions, *µ*_max_ values ranged from 0.003 (strong light intensity µmol photons m^−2^ s^−1^) to 0.113 day^−1^, reflecting greater variability and occasional reductions in kinetic efficiency, particularly above ∼700 µmol m^−2^ s^−1^ (SI, Fig. 9S). These patterns are consistent with previous reports showing that PCC 6803 tolerates high irradiance but does not proportionally increase *µ*_max_ beyond saturated light concentration.^47^ The enhanced *X*_max_ values under high light likely resulted from increased initial biomass and improved internal shading, a phenomenon documented in cyanobacterial dense cultures.^48^ Thus, high light increased final culture yields but offered no kinetic advantage compared to the moderate-light optimum.

Across all illumination categories, Monod reproduced biomass trajectories with high fidelity (adjusted *R*^2^ > 0.95; residual distributions in the SI). The *µ*_max_ values spanned a narrow but biologically meaningful range (0.081–0.118 day^−1^), with nearly a 50% increase between the poorest low-light and most favourable moderate-light conditions (Table 1). Moderate light (421–598 µmol m^−2^ s^−1^) consistently yielded superior *µ*_max_ values, suggesting that this regime corresponds most closely to the physiological optimum for PCC 6803 under nutrient-replete batch conditions. High-light conditions (>600 µmol m^−2^ s^−1^) increased total biomass but not growth rate, indicating early saturation of light-harvesting efficiency. These kinetic parameters form the quantitative foundation for subsequent continuous-cultivation modelling. The moderate-light *µ*_max_ (0.118 day^−1^) and corresponding *X*_max_ values provide the best estimate of the intrinsic growth ceiling of the wild strain, while the low-light and high-light margins delineate physiologically realistic lower and upper operational boundaries.

**Table 1:** Summary of Monod kinetic parameters for batch cultures under three illumination regimes

Illumination category	PPFD range (µmol m^−2^ s^−1^)	Representative *µ*_max_ (day^−1^)	Range across replicates	Representative *X*_max_ (g L^−1^)	Range across replicates
Low light	99–287	0.106 ± 0.002	0.08–0.118	2.27 ± 0.03	1.08–2.27
Moderate light	421–598	0.116 ± 0.003	0.05–0.116	1.96 ± 0.02	0.87–1.99
High light	600–1430	0.064 ± 0.002	0.003–0.113	1.01 ± 0.02	0.58–1.01

### 3.5. Implications and future perspectives

The integrated experimental and modelling analyses presented in Sections 3.2–3.4 have direct implications for the design of energy-efficient and environmentally sustainable photobioprocesses. Although photosynthetic microbial platforms inherently align with green chemistry principles by converting CO_2_ and light into biomass and valuable products, their true sustainability depends on how effectively photon, nutrient, and energy inputs are transformed into valuable outputs. The present study provides quantitative boundaries that enable continuous cyanobacterial cultivation to operate within a regime that maximizes photonic efficiency, minimizes energy waste, and prevents biomass loss through washout or photostress. For instance, the multi-factor kinetic model demonstrates that the combined influence of light, temperature, and dilution rate produces a far more constrained operational space than traditional light- or nutrient-limited models predict. By integrating these parameters into a single mechanistic framework, we provide a tool for minimising resource waste. For example, the model shows that reducing dilution rate slightly (*e.g.*, from 0.14 to 0.12 d^−1^) may decrease biomass productivity by only ∼5–8%, but dramatically increases tolerance to fluctuations in temperature or illumination, thereby preventing washout-induced culture collapse. This is consistent with long-established photobioreactor analyses showing that productivity peaks only within a narrow photon-use window, and that illumination beyond metabolic saturation results in wasted radiant energy and reduced system efficiency.^49^ Such model-informed decision-making supports the green chemistry principle of inherently safer, more stable process design, reducing the need for corrective interventions, reinoculation, or emergency shutdowns.^50^

The quantitative insights developed here enable the translation of biological performance into environmentally relevant metrics, particularly energy efficiency and CO_2_ sequestration. Using the experimentally validated volumetric productivity measured in the optimised continuous regime (0.07–0.125 g L^−1^ day^−1^), a 120 L mini-pilot reactor would generate approximately 8.4–15.0 g biomass day^−1^. Assuming a conservative higher heating value of 20–22 kJ g^−1^ for cyanobacterial biomass,^51^ this corresponds to 176–315 kJ day^−1^ (0.049–0.087 kWh day^−1^) of chemical energy stored through photosynthesis. Although modest in absolute magnitude, the relevance becomes clearer when compared to the energy requirement for light illumination. At PPFD levels of 500–700 µmol photons m^−2^s^−1^, the daily incident radiant energy is approximately 2.6–3.1 kWh m^−2^ day^−1^, whereas operation at 700–800 µmol photons m^−2^s^−1^ would increase energy demand to 3.7–4.2 kWh m^−2^ day^−1^ without measurable gains in biomass.^49^ Even before accounting for electrical inefficiencies and power-supply losses, this illustrates that operating beyond the biologically optimal photon flux results in an additional 30–40% energy burden with no productivity benefit. When scaled to the illuminated surface area of a 120 L photobioreactor (typically 0.15–0.25 m^2^ for flat-panel geometries), the corresponding daily lighting energy requirement would fall in the range of 0.4–0.8 kWh, while the pumping and mixing load would contribute an additional 0.02–0.05 kWh day^−1^. Against this operational energy input, the chemical energy stored in the biomass (0.049–0.087 kWh day^−1^) corresponds to an apparent energy recovery of 6–15%. While not competitive as an energy-harvesting strategy, this demonstrates that continuous cyanobacterial cultivation can maintain a reasonably balanced energy profile when operated within the narrow, model-defined optimum. More importantly, these values highlight a key green-chemistry insight, *i.e.*, energy-neutral or energy-balanced phototrophic systems are unattainable when illumination exceeds metabolic saturation, reinforcing the value of mechanistically informed illumination control.

Moreover, the environmental relevance becomes clearer when biomass production is expressed as CO_2_ fixation. Adopting the widely used stoichiometric factor of ∼1.8 g CO_2_ fixed per gram of microalgal biomass,^52^ the same 120 L system would sequester 15.6–27.6 g CO_2_ day^−1^, equivalent to 5.7–10.1 kg CO_2_ annually under the optimised window identified in this study. While these values do not approach industrial carbon-capture scales, they are significant for a 120 L unit operating under laboratory-scale conditions. More importantly, they demonstrate that each gram of fixed CO_2_ is achieved under a photon-use regime that deliberately avoids excessive irradiation, showing that sustainable carbon removal and low-waste operation can be achieved simultaneously when illumination, hydrodynamics, and temperature are tuned according to the mechanistic boundaries established here.

These findings open the way for the development of truly green phototrophic technologies. The narrow operating window revealed here suggests clear opportunities for model-predictive process control, where real-time sensing of PPFD, temperature, and growth rate informs automated adjustments to dilution rate or shading intensity. Such strategies could maintain cultures within the high-efficiency zone despite fluctuations in ambient light or reactor temperature. The kinetic model also provides a foundation for re-parameterisation in larger photobioreactor geometries, enabling predictions of scale-dependent stressors such as light attenuation, internal gradients, or diurnal thermal swings—factors that become critical in outdoor or solar-driven systems. Finally, quantifying biomass-energy content and CO_2_ capture provides a blueprint for integrating cyanobacterial systems into circular manufacturing frameworks, where photobioreactors can serve as both bioproduct generators (pigments, protein-rich biomass, bioplastics) and low-energy carbon sinks.

## 4. Conclusions

This study demonstrates that continuous cultivation of *Synechocystis* sp. PCC 6803 can achieve high stability, predictable performance, and environmentally meaningful productivity when operated within a narrow, mechanistically defined window. By integrating controlled photophysiology, long-term continuous experimentation, single- and multi-factor analysis, and a revised kinetic framework grounded in batch-derived Monod parameters (*µ*_max_ = 0.081–0.118 day^−1^), we define a precise operational regime—500–700 µmol photons m^−2^ s^−1^, 32–34 °C, and *D* = 0.12–0.14 d^−1^—that maximises photon utilisation while avoiding washout, dilution-induced stress, and photoinhibition. The kinetic characterisation further establishes that moderate illumination produces the physiological growth ceiling for the wild strain and provides parameter constraints essential for predicting continuous-mode behaviour. Translating these biological insights into sustainability metrics shows that even a 120 L mini-pilot system can convert low-grade photon energy into 176–315 kJ day^−1^ of chemical energy and fix 15–28 g CO_2_ day^−1^ with minimal resource input. The operating window quantified here should be interpreted as a representative, well-characterised case for *Synechocystis* sp. PCC 6803 in a calibrated flat-panel photobioreactor rather than a universally transferable solution. While the specific numerical boundaries are system- and strain-dependent, the central finding, that continuous phototrophic systems operate within a narrow, coupled multi-parameter window, is expected to be broadly applicable.

## Author contribution

Mohammad Redwanur Rahman (MRR): conceptualisation, methodology, investigation, formal analysis, data curation, visualisation, writing – original draft, writing – review & editing. Md Tabish Noori: data curation, writing – original draft, writing – review & editing, manuscript formatting, submission handling. Klaus Hellgardt: supervision, conceptualisation, review of experimental design, validation, writing – review & feedback.

## Conflicts of interest

The authors are co-founders of Brilliant Dyes. The authors declare that there are no conflicts of interest related to this work.

## Supplementary Material

RA-016-D5RA09945E-s001

## Data Availability

All processed datasets supporting the findings of this study are provided in the supplementary information (SI). Supplementary information: Table S1 detailing the modified BG-11 medium composition; a dedicated section outlining energy, carbon, and efficiency calculations; reactor setup schematics for the batch FMT150 photobioreactor system (Fig. S1); OD_680_–dry cell weight calibration data (Fig. S2); spectral characterisation and calibration of all four LED modules used in the FP-PBR experiments, including intensity comparisons and calibration slopes (Fig. S3 and S4); stock culture validation and UV-vis characterisation of *Synechocystis* sp. PCC 6803 (Fig. S5); long-term continuous cultivation performance over 90 days (Fig. S6); and complete kinetic model fits under low, moderate, and high illumination regimes (Fig. S7–S9). Together, these materials provide all numerical datasets, calibration relationships, model parameters, and extended experimental results necessary to reproduce the analyses and figures presented in the manuscript. Raw photobioreactor operational logs (including OD, pH, temperature, PPFD, and full time-resolved datasets) are not included in the SI due to file size but are available from the authors in Excel format upon reasonable request. See DOI: https://doi.org/10.1039/d5ra09945e.
