# Supplementary material for: Photonic-chemostat engineering for efficient continuous cultivation of cyanobacteria
Source: RSC Adv. 2026 Feb 25;16(12):11036–48. doi: 10.1039/d5ra09945e (PMC12933868; doi:10.1039/d5ra09945e)
Supplement: RA-016-D5RA09945E-s001 [file RA-016-D5RA09945E-s001.pdf]

Supplementary Information (SI) for RSC Advances.
This journal is © The Royal Society of Chemistry 2026

Photonic–chemostat engineering for efficient continuous cultivation of cyanobacteria

Supplementary information

S1. BG11 Medium Composition and Preparation Protocol

Synechocystis sp. PCC 6803, a cyanobacterium widely used for basic research, is usually cultivated in a synthetic medium, BG-11**.** It is still poorly understood what limits the growth of this organism in batch and continuous cultures in BG-11. Sulfate is the limiting nutrient in BG-11, as well as in BG-11-Prolonged Culture. In this PhD research, we used a modified BG-11 (based on recent research and advancement from Professor Patric Jones group at the Department of Lice Sciences, Imperial College London) for *Synechocystis* sp. PCC 6803 as shown in Table 1S.

Table 1S: Modified BG-11 medium closely aligns with the recipe of standard BG-11 media, which was developed by Stainer et al (Stanier et al., 1971).

	**Chemicals**	**Product Source and Product Number**	**Per 500ml**
(1)	$NaNO_3$	Sigma Aldrich; 237213 and S5022	75.0 g
(2)	K_2HPO_4	Sigma Aldrich; P5504	2.0 g
(3)	$MgSO4.7H_2O$	Sigma Aldrich; M5921	3.75 g
(4)	$CaCl_2.2H_2O$	Sigma Aldrich; C3306	1.80 g
(5)	Citric acid *	Sigma Aldrich; 251275	0.30 g
(6)	Ammonium ferric citrate green *	SAFC RES20400-A2702X	0.30 g
(7)	$EDTANa_2$	Fluka Analytical; 433802	0.05 g
(8)	Na_2CO_3	Sigma Aldrich; S7795 and 71345	1.00 g
(9)	Pentahydrate Na-thiosulfate (extra)	Sigma Aldrich; S8503	47.0 g
(10)	**Trace metal**		**Per litre**
	H_3BO_3	Sigma Aldrich; 9645 and 197335	2.86 g
	$MnCl_2.4H_2O$	Sigma Aldrich, M3635 and Fluka, 63535	1.81 g
	$ZnSO4.7H_2O$	Sigma Aldrich; Z4750	0.22 g
	$Na_2MoO_4.2H_2O$	Fluka Analytical; 71756	0.39 g
	$CuSO_4.5H_2O$	Sigma Aldrich; C3036 and C8027	0.08 g
	$Co(NO_3)_2.6H_2O$	Sigma Aldrich; 239267 and 203106	0.05 g

Stock solutions 1-9 have been taken at 10.0 ml per litre, and stock solution 10 has been added at 1.0 ml per litre. After the stock solution is added, DI water is brought to 1 litre and autoclaved before use. Following that, adjusted the pH to 7.1 with 1M NaOH or HCl. For agar, we added 15.0 g per litre of Bacteriological Agar (Oxoid L11). Then autoclaved at 15 psi for 15 minutes.

Due to precipitation, stocks 5 and 6 are not autoclaved, but instead are used with a sterile syringe filter to add to the autoclaved media under the airflow cabinet.

S2. Energy, Carbon, and Efficiency Calculations

S2.1 Photon energy calculation

Energy per photon:

$$E_{\text{photon}} = \frac{hc}{\lambda}$$

where

$h = 6.626 \times 10^{-34}$ J\cdotps,

$c = 3 \times 10^{8}$ m s^{-1},

$\lambda = 550$ nm.

Daily photon energy (per m^2):

$$E_{\text{day}} = PPFD \times 86400 \times \frac{E_{\text{photon}}}{N_A}$$

S2.2 Biomass productivity

$$P_{\text{vol}} = D \cdot X$$

where

D= dilution rate (day^{-1}),

X= steady-state biomass (g L^{-1}).

S2.3 Biomass energy storage

With biomass HHV (20–22 kJ g^{-1}):

$$E_{\text{biomass}} = P_{\text{vol}} \times V \times HHV$$

S2.4 CO_2 sequestration

Using 1 g biomass = 1.8 g CO_2:

$$F_{CO_2} = 1.8 \times P_{\text{vol}} \times V$$

S2.5 Photosynthetic efficiency (PE)

$$\eta_P = \frac{E_{\text{biomass}}/A}{E_{\text{day}}}$$

where A is illuminated area.

S2.6 Energy Return on Operational Energy Invested (EROOI)

$$EROOI = \frac{E_{\text{biomass}}}{E_{\text{electrical}}}$$

where

$E_{\text{electrical}}$= (LED + pumps + aeration) × 24 h.

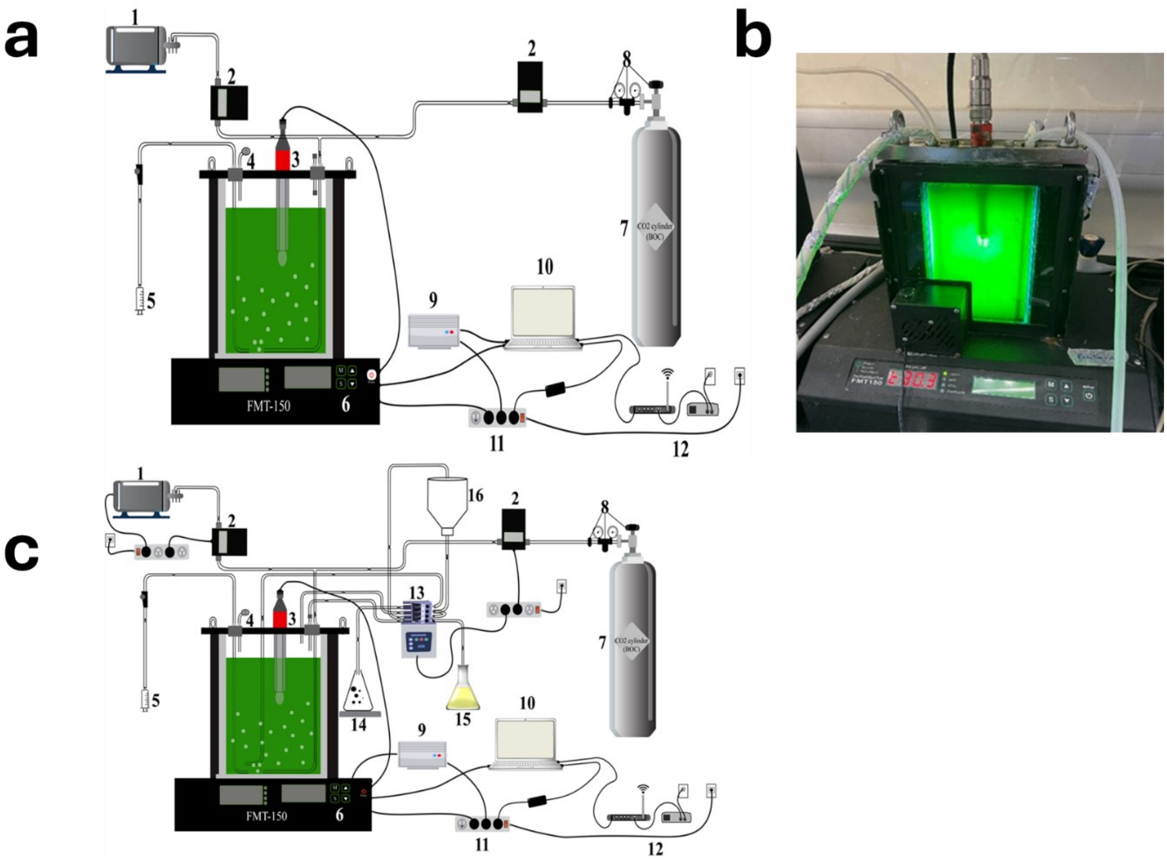

Fig. 1S: (a) Layout of the setup of the batch experimental trial in FMT150 Photobioreactor. (Here, 1. Gas compressor, 2. Mass flow controller, 3. Temperature/pH sensor, 4. Air pressure release through a sterile filter, 5. Sampling syringe, 6. Manual control panel, 7. CO_2 cylinder (BOC), 8. Stopper for CO_2 flow control, 9. FMT150 power supply, 10. Software control of FMT150, 11. Power source, 12. Internet connection for remote control of the experiment with the FMT150 software, (b) the real test PBR, and (c) Chemostat system design for the continuous separation of octyl acetate from cyanobacteria. (Here, 1. Gas compressor, 2. Mass flow controller, 3. Temperature/pH sensor, 4. Air pressure release through a sterile filter, 5. Sampling syringe, 6. Manual control panel, 7. CO_2 cylinder (BOC), 8. Stopper for CO_2 flow control, 9. FMT150 power supply, 10. Software control of FMT150, 11. Power source, 12. Internet connection for remote control of the experiment with FMT150 software, 13. Peristaltic pump, 14. Waste, 15. Media, 16. Separator).

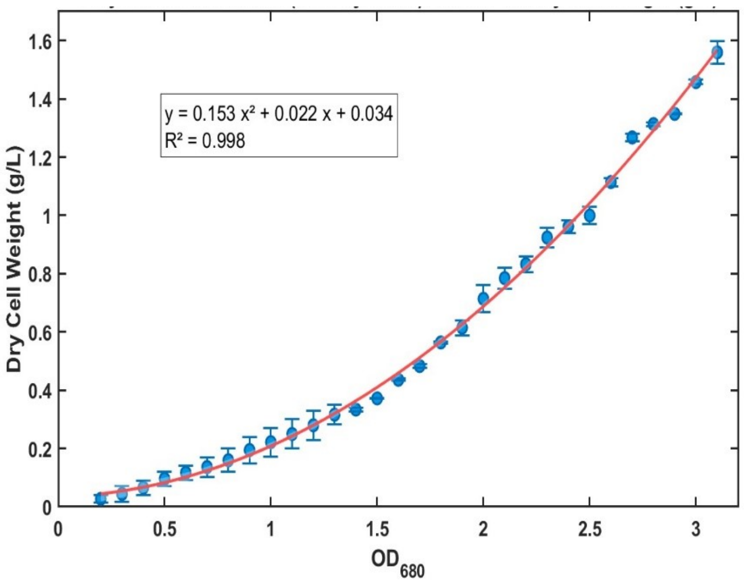

Fig. 2S: Calibration of OD680 vs Dry Cell Weight of Wild *Synechocystis* sp. PCC 6803 from low to high OD. 10 ml of each sample with duplicates has been oven dried at 50 °C for overnight (8 hours) to get the dry biomass weight, which was used for this calibration curve and follow-up experiments.

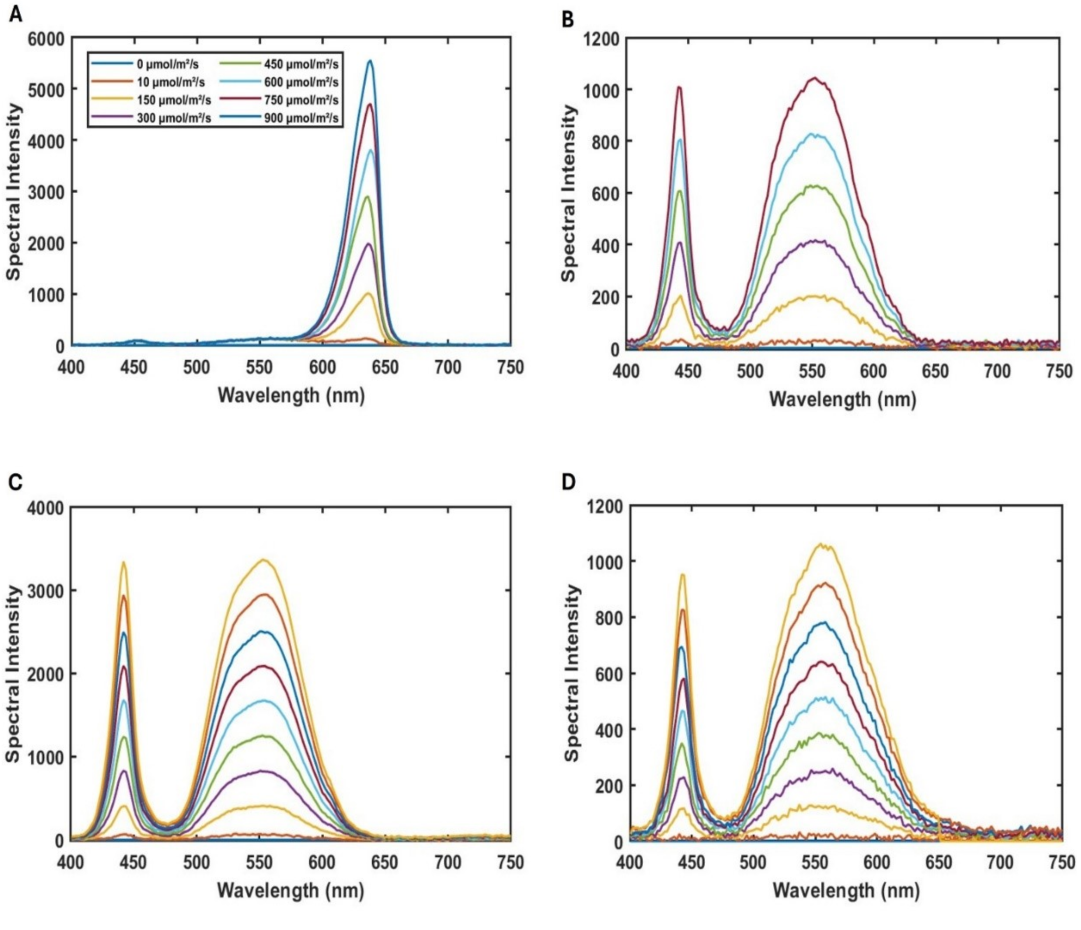

Fig. 3S: Comparison among the spectral intensity of 4 different LED modules FP-PBRs (we assigned different conditions as PBR-1-4 here) used in the customised designed batch trials

at different wavelengths (400 to 750 nm) (a) LED module dominant Actinic Red LED illumination (b-d) with Actinic White LED illumination.

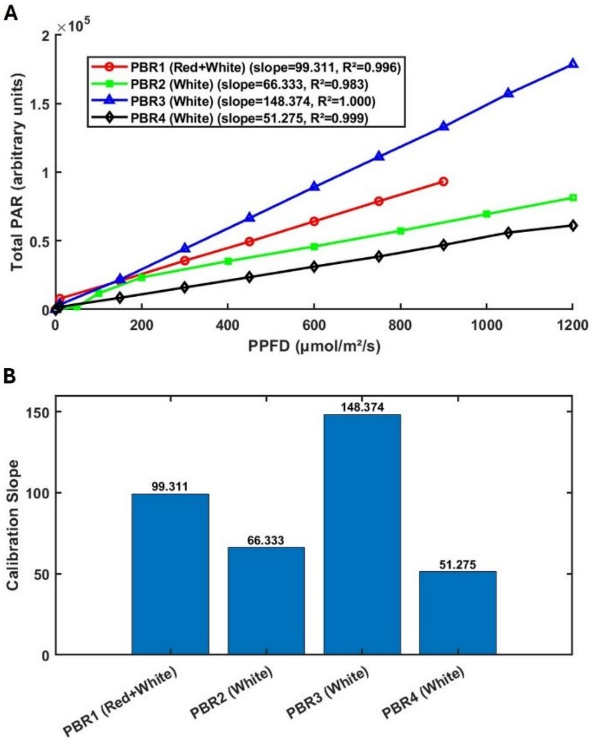

Fig. 4S: (A) Calibration curve of 4 different LED modules in PBRs used in this research for designed experiments in batch and continuous trials. (B) Calibration slopes of PBR with a significant difference from the standard reference LED lamps intensity.

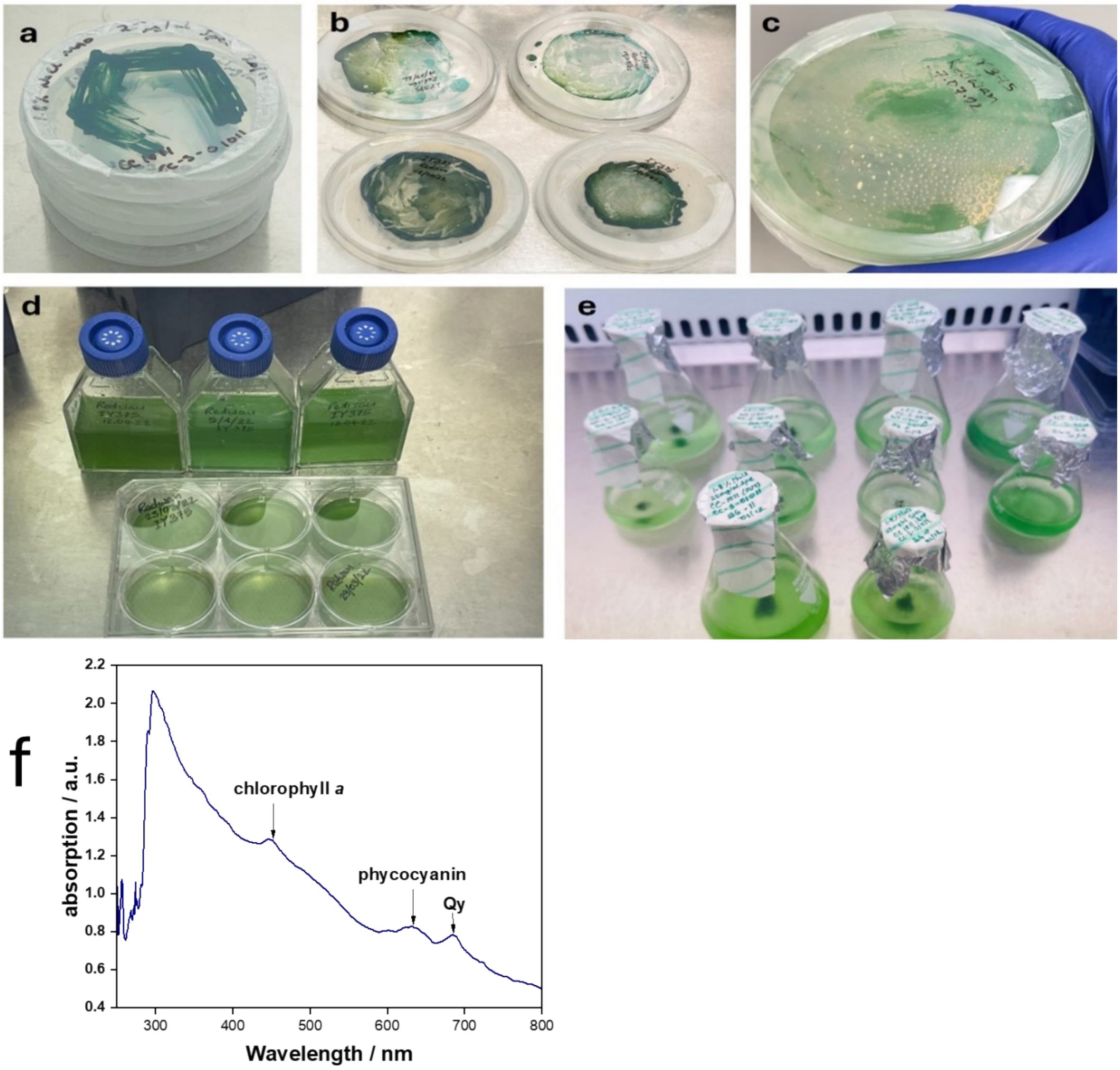

Fig. 5S: *Synechocystis* sp. PCC 6803 stock culture (a-c) BG11 agar plate, (d-e) BG11 suspended, and (f) UV-Vis spectrum of suspended PCC 6803

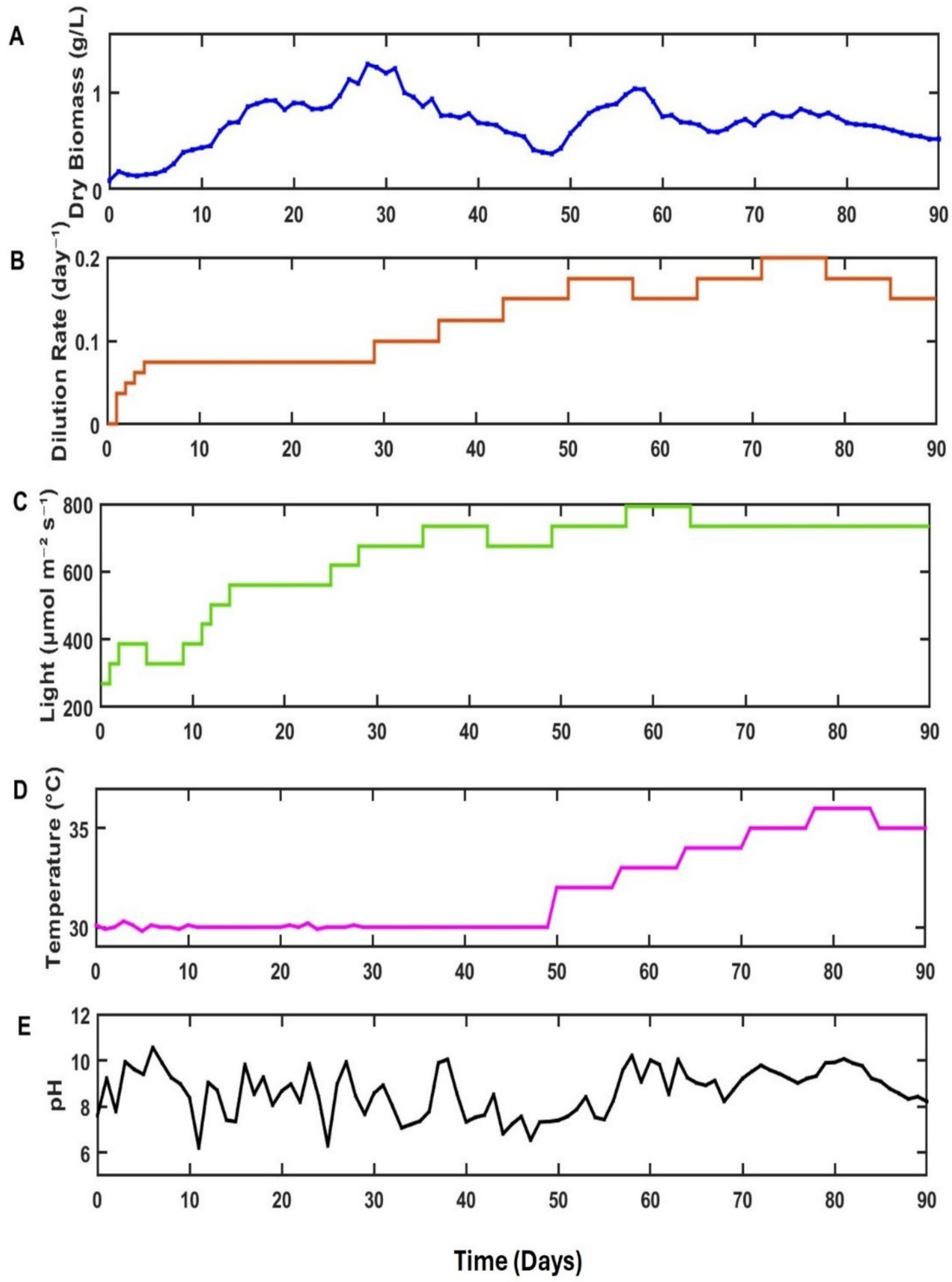

Fig. 6S: Continuous cultivation of wild PCC 6803 over 90 days. (A) Biomass (g/L) estimated from OD_{680} remained stable during most of the run, with declines corresponding to washout events. (B) Dilution rate started at 0.0375 day^{-1} and was progressively increased. Rates ≥0.175 day^{-1} resulted in repeated washout, indicating this value as the upper stability limit for this system. (C) Light intensity was gradually raised from 270 to ~790 μmol photons m^{-2} s^{-1} to test photophysiological limits; intensities above ~730 μmol photons m^{-2} s^{-1} offered no further productivity gain, defining the practical light threshold. (D) The temperature was held at 30 °C for the first 49 days, then increased stepwise to 36 °C. Growth remained stable up to ~34–35 °C, but performance declined at higher temperatures. (E) pH fluctuated between 6 and 10.5 due to CO_2 dosing limitations and metabolic activity, but generally remained within the acceptable physiological range for *Synechocystis*, with brief excursions above pH 9.5 when CO_2 supplementation was insufficient.

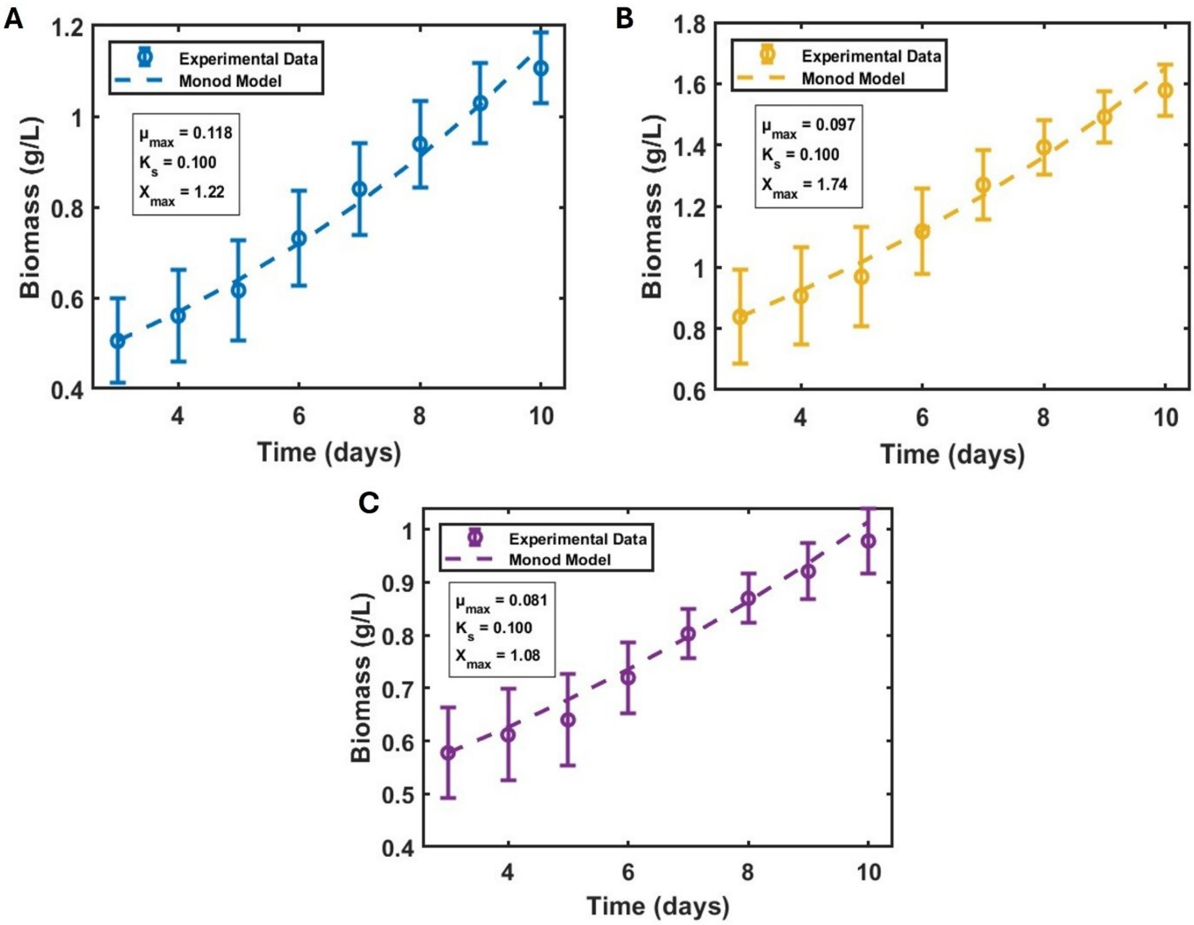

Fig. 7S: Kinetic Models at Low Light Intensity (99 - 287 μmol photons m^{-2} s^{-1} Actinic White conditions: (a) 99μmol photons m^{-2} s^{-1}, (b) 287 μmol photons m^{-2} s^{-1}, (c) 253 μmol photons m^{-2} s^{-1}.

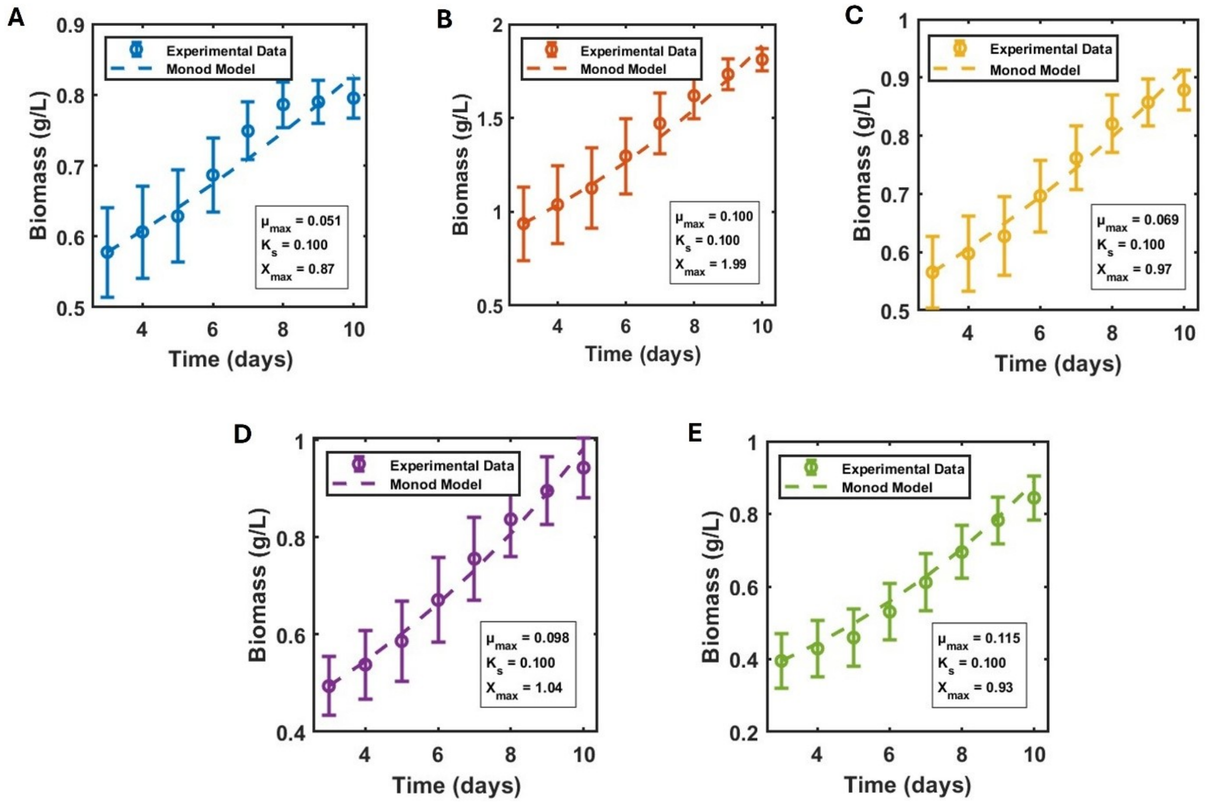

Fig. 8S: Kinetic Models at moderate Light Intensity (300 - 598 µmol photons m^{-2} s^{-1} Actinic White conditions (a) 598 µmol photons m^{-2} s^{-1} Actinic White (b) 598 µmol photons m^{-2} s^{-1} Actinic Red and White (c) 598 µmol photons m^{-2} s^{-1} Actinic White (d) 584 µmol photons m^{-2} s^{-1} White and (e) 421 µmol photons m^{-2} s^{-1} White.

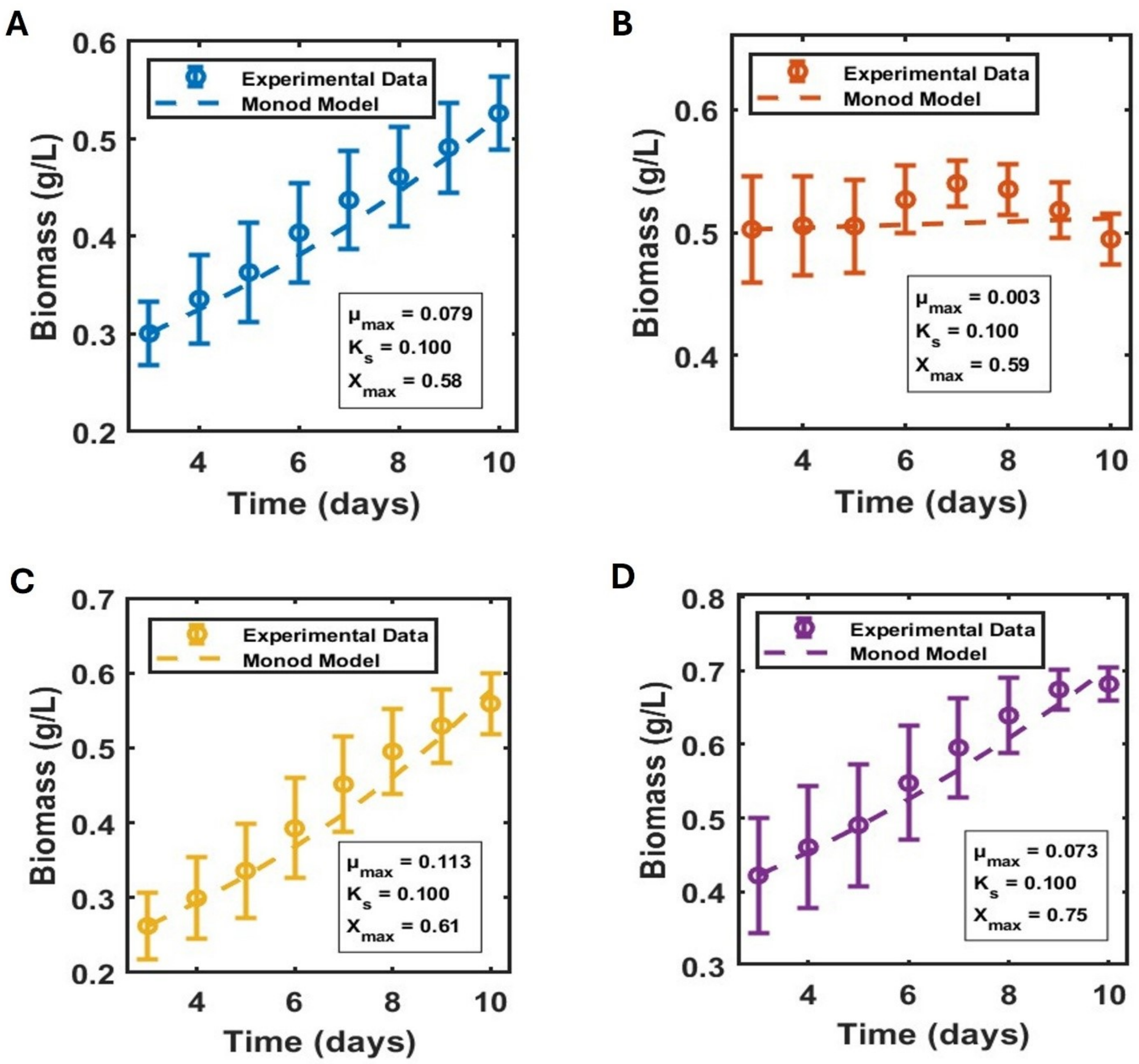

Fig. 9S: Kinetic Models at high Light Intensity (600 – 1430 µmol photons m^{-2} s^{-1}) (a) 953 µmol photons m^{-2} s^{-1} Actinic Red and White, (b) 1430 µmol photons m^{-2} s^{-1} Actinic White LED, (c) 953 µmol photons m^{-2} s^{-1} Actinic Red and White and (d) 600 µmol photons m^{-2} s^{-1} Red and White.
